# Genomic Analysis as the First Step toward Personalized Treatment in Renal Cell Carcinoma

**DOI:** 10.3389/fonc.2014.00194

**Published:** 2014-07-25

**Authors:** Zofia Felicja Bielecka, Anna Małgorzata Czarnecka, Cezary Szczylik

**Affiliations:** ^1^Department of Oncology with the Laboratory of Molecular Oncology, Military Institute of Medicine, Warsaw, Poland; ^2^Postgraduate School of Molecular Medicine, Medical University of Warsaw, Warsaw, Poland

**Keywords:** genomics, personalized treatment, prognostic and predictive biomarkers, high-throughput techniques, genome-wide analysis, translational research, renal cell carcinoma, tumor heterogeneity

## Abstract

Drug resistance mechanisms in renal cell carcinoma (RCC) still remain elusive. Although most patients initially respond to targeted therapy, acquired resistance can still develop eventually. Most of the patients suffer from intrinsic (genetic) resistance as well, suggesting that there is substantial need to broaden our knowledge in the field of RCC genetics. As molecular abnormalities occur for various reasons, ranging from single nucleotide polymorphisms to large chromosomal defects, conducting whole-genome association studies using high-throughput techniques seems inevitable. In principle, data obtained via genome-wide research should be continued and performed on a large scale for the purposes of drug development and identification of biological pathways underlying cancerogenesis. Genetic alterations are mostly unique for each histological RCC subtype. According to recently published data, RCC is a highly heterogeneous tumor. In this paper, the authors discuss the following: (1) current state-of-the-art knowledge on the potential biomarkers of RCC subtypes; (2) significant obstacles encountered in the translational research on RCC; and (3) recent molecular findings that may have a crucial impact on future therapeutic approaches.

## Introduction

Among solid urological tumors, renal cell carcinoma (RCC) has the highest death rate. About 40% of patients with this condition do not survive. Moreover, symptomatic metastases are observed in 33% of the cases at diagnosis. The morbidity of RCC constantly increases by about 1.5–5.9% annually, but due to the development of new non-invasive diagnostic methods, this increase is not rapid. However, this tumor remains the 10th most common in men and 14th most common in women. Consequently, it is still perceived as a serious disease that can affect the entire population, also children (1).

Kidney cancers are mostly of sporadic origin, but some patients are genetically predisposed, suffering from von Hippel-Lindau (VHL) syndrome, which is connected with the loss of the *VHL* suppressor gene function (*VHL* follows an autosomal dominant hereditary pattern) (2). Around 40–60% of patients with mutated *VHL* suffer from clear cell renal cell carcinoma (ccRCC). Other RCC subtypes are comprised (according to the World Health Organization system) of the following: (1) renal oncocytoma; (2) papillary renal cell carcinoma (PRCC); and (3) chromophobe renal cell carcinoma (chRCC). They may also comprise recently recognized rare malignancies, such as (4) collecting ducts of Bellini renal cell carcinoma (cdRCC); (5) renal medullary carcinoma; (6) renal carcinoma associated with the translocation of locus 11.2 on the short arm of the X chromosome; and (7) mucinous tubular spindle cell carcinoma (3, 4).

von Hippel-Lindau syndrome was first shown by German ophthalmologist Eugen von Hippel, who described angiomas in the eye in 1904 (5). Subsequently, Arvid Lindau described the angiomas of the cerebellum and spine in 1927 (6, 7). The term “VHL disease” was first used in 1936; however, its use became common only in the 1970s. Since 1926, almost 400 cases of VHL disease have been reported in the literature (4). VHL mutation was observed in renal cell carcinoma *inter alia* by Seizinger et al. in 1988. Their article was published in Nature (3). The authors confirmed that the disorder followed an autosomal dominant inheritance pattern and that it predisposed to cancerogenesis.

Later on, VHL gene was shown to be located on the short arm of the third chromosome (3p25), which also mapped the locus encoding *RAF1* (v-raf-1 murine leukemia viral oncogene homolog). As sporadic forms of RCC have previously been associated with the loss of specific regions of chromosome 3p, this information was crucial (4). Additionally, the active form of the *RAF1* oncogene has a significant impact on the protein synthesis in the RAS/mitogen-activated protein kinase (MAPK) signaling pathway (8). The latter transmits chemical signals from outside the cell to its nucleus. It also controls cell growth, proliferation, differentiation, migration, and apoptosis (8).

In 1990, Hosoe et al. performed genetic linkage analysis on families with *VHL*. This complex study took almost 10 years to complete and it resulted in the fundamental identification of many mutations in 1998 (9). However, the first signs that *VHL* may be connected with kidney cancers in general were reported in 1979 by Cohen et al., who published a report about a single family, some members of which were presented to have bilateral multifocal ccRCC connected with the translocation of chromosome 3 (9). This gene was attributed only to ccRCC, since *VHL* mutations are not found in papillary, chromophobe, collecting duct, or medullary renal cancer (10–13).

It is worth noting that VHL protein is a substrate of E3 ubiquitin protein ligase complex. It is also the main regulator of the hypoxia inducible factor (HIF) (mainly 1-α and 2-α; the role of HIF3-α is unknown). E3 binds α subunits, leading to their ubiquitination and further degradation (14, 15). The role of HIF subunits in kidney cancer will be described in detail later.

Up to 40% of sporadic ccRCCs have a wild-type form of *VHL* (non-mutated). This leads to the assumption that ccRCC is a disease of many mutations and is, therefore, highly heterogeneous (16). Renal cell carcinoma is now perceived as a heterogeneous cancer in general (17). There are many known RCC subtype-dependent genetic alterations up to date. These include the *SET* domain containing protein 2 (*SETD2*), *PBMR1*, and *BAP1* genes, which will be mentioned and described in later chapters.

The increasing frequency of conducting genetic research shows that a wide variety of methods concerning the fast identification of genetic and epigenetic RCC disorders exist. Currently, researchers use more precise methods, especially comparative genomic hybridization (CGH), high-resolution CGH and array CGH, as well as whole-genome arrays. Only detailed genomic analyses allow the complex description of genetic alterations in renal cell carcinoma and the linking of tumorigenic changes in the kidney with genetic alterations on a global level. This review shows why high-throughput techniques used in genomic studies may become the first step toward personalized treatment in renal cell carcinoma (16, 17).

In this review, authors aim to prove that RCC is a heterogeneous tumor and harbors several mutations characteristic only for each particular subtype of this cancer. This will be described in the first chapter. Secondly, it will be shown that RCC is a potent target to seek for potential diagnostic, prognostic, and predictive biomarkers, which may solve many problems that currently clinicians have to face. The authors will also present some results in this matter, which are promising and may lead to further classification of specific molecular RCC patterns. Furthermore, the authors will present evidence that those mutations are significant components of crucial signaling pathways leading to cancerogenesis and metastases. On the other hand, the authors will raise significant critical questions concerning renal cell carcinoma and they will seek for an answer, whether genomic analysis really is more promising in RCC than in other cancers and why it is necessary to conduct more experiments on the genomic level in this field. Finally, after discussing the current state-of-the-art in RCC personalized treatment, the authors will conclude on to which extent RCC personalized treatment is possible and whether genomic studies still have potential to broaden the knowledge of molecular oncologists and in the end clinicians as well, to implement it in everyday life.

## RCC is a Genomic Disease and it Harbors Unique Mutations Dependent on Its Histological Subtype

According to Larkin et al., renal cell carcinoma is widely defined as a “constellation of malignancies of different histological subtypes arising from renal parenchyma” (16). This means that patients with RCC exhibit different sets of mutations depending on what histological subtype of RCC they suffer from.

### Clear cell RCC

The most common RCC subtype, ccRCC possesses several distinct genetic alterations. These include the previously mentioned loss of function of the VHL tumor suppressor gene, inactivation of the *SET* domain containing protein 2 (*SETD2), KDM6A, KDM5C* – both lysine (K)*-*specific demethylases – and polybromo1 (*PBRM1*). These genes are mentioned together because of their common function; they are all chromatin remodeling complex genes (15). Chromatin remodeling means dynamic chromatin modifications in its construction, which enables various transcription factors to be selectively attached to the condensed structure of DNA. This is achieved through several various histone modifiers (17). Knowledge regarding this matter has been acquired through the large-scale sequencing of ccRCC tumors. Polybromo1 has been found to be mutated in ~40% of cases, being the second most mutated gene in ccRCC after *VHL*. Knowing this, it is easier to target this molecule if it is a specific target in the ccRCC treatment (17).

More recently, *BRCA* related-protein 1 (*BAP1*) has been reported in 4% of 98 ccRCC samples (18). In addition, *SETD2* (mutated in 8% of cases) was found to be located on the short arm of the third chromosome (like *PBMR1*) close to the specific locus of *VHL*. *SETD2* was a histone methyltransferase and *BAP1* was its deubiquitinase. Both were involved in the remodeling of the chromatin. The relation between these three genes has been studied by Hakimi et al. in the previous year (19). In the study, targeted sequencing was conducted in 185 ccRCC cases. Apart from the significant percentage of mutations of the three genes mentioned above, *KDM5C* was also shown to be mutated in 8% of the cases (19). A study of 145 patients with developed ccRCC reported that in those harboring *BAP1* and *PBMR1* mutations, the overall survival (OS) rate was significantly shorter than in patients harboring only *PBMR1* mutation. It is worth noticing that those results have been validated in a cohort from the Cancer Genome Atlas study (20, 21).

Among other genes, hypoxia-inducible factors are significant in ccRCC. They are inhibited by the functional protein of the *VHL* gene under normoxic conditions via the polyubiquitination process (it is known that it concerns HIF-1α). If lack of oxygen occurs (hypoxia is a natural condition in most tumors), HIF-1α is not degraded; therefore, it accumulates and binds to a stable β-subunit of HIF-1. In consequence, *HIF* genes are activated, regulating angiogenesis. As a result, new vessels are excessively formed to “feed” the tumor. This phenomenon is characteristic of ccRCC and has been proven by various genomic research (22–24).

Taking these data together, it may be assumed that ccRCC possesses its own distinctive mutations in its genetic profile. In the study of Zhang et al., wherein cytogenetic profiles of four RCC tumor subtypes have been compared, −3p, +5q, and −8p have been perceived as unique to ccRCC tumors (25). What is more, research by Pei et al. (26) using both classical cytogenetics as well as microarray-based genomic copy-number analysis has revealed several similar rearrangements in ccRCC. SNP-based arrays performed by this scientific group showed the deletion of 3p in 16 out of 20 tumors (80% cases) (26).

Summing up, *VHL* gene is a key factor, mostly because of the following reasons: (a) it often predisposes patients with *VHL* syndrome to develop hereditary ccRCC; (b) it is the most common alteration in sporadic ccRCC; and (c) it is found even in small ccRCCs at early diagnosis (26).

### Papillary RCC

The hereditary form of PRCC (HPRCC, type I PRCC) also has its specific proto-oncogene: *MET*. Schmidt et al. first performed genetic linkage analysis on this type of cancer in 1997. Mutations of *MET* proto-oncogene have been noticed in its tyrosine kinase domain, which has classified HPRCC for targeted therapy against *MET* and vascular endothelial growth factor (*VEGF*) receptors (27). Two years later, the same scientific group reported that hereditary PRCC was characterized by multiple, bilateral papillary renal carcinomas. They also screened a large group of sporadic papillary renal carcinomas and many other solid tumors for various mutations in *MET* proto-oncogene. Summarizing these and the previous results, they have presented such mutations in 17 out of 129 sporadic papillary renal carcinomas, but not in other solid tumors. What was striking was that even in 1999, molecular modeling studies suggested the importance of activating mutations. It was known that such phenomena tended to facilitate transition to the active form kinases, in this case *MET* kinase, leading to the formation of a different histological RCC subtype (28).

Type II papillary kidney cancer has been described in literature along with “hereditary leiomyomatosis renal cell carcinoma (HLRCC),” which also has its characteristic gene – the fumarate hydratase coding gene. If a specific mutation disables the *FH* gene, the Krebs cycle would be modified, favoring the excessive synthesis of fatty acids, which in turn promotes tumor growth (Warburg effect) (29).

### Chromophobe RCC

A hereditary form of chromophobe renal cancer also exists. It is called the Birt-Hogg-Dubé (BHD) disease. In this condition, patients usually develop bilateral, multifocal renal tumors, fibrous folliculomas, and/or pulmonary cysts. This type of disease is more complex than other kidney cancers. Patients with VHL develop ccRCC, while patients with HPRC develop type 1 papillary kidney cancer. However, patients suffering from BHD tend to develop a spectrum of renal tumor subtypes, including chromophobe, clear cell, oncocytomas, or hybrid oncocytic tumors (30). BHD has turned out to be connected with the folliculin-coding gene (FLCN) mutation. Generally, it is perceived as a tumor suppressor as its several types of mutations lead to various tumor formations. Vocke et al. have identified folliculin mutation in about 70% of patients in the research probe (31). It should be noted that this gene belongs to the LKB1/AMPK signaling pathway. The protein encoded by FLCN leads to several processes, including the binding of protein FNIP1, FNIP2, and gamma subunit of AMPK, an AMP-activated protein kinase. As the LKB1 tumor suppressor kinase is an activator of the AMPK, it forms a number of cellular energetic levels by favoring either catabolic or anabolic processes. It is known that this pathway controls, among others, cell polarity. The molecular link between such polarity and metabolism may result in a stress-response protective mechanism, which in turn leads to tumor suppression during evolution (32–34).

The proper diagnosis of kidney cancer subtypes is becoming more and more complicated and difficult to achieve after the recognition of novel RCC subtypes. Owing to this, several researches in oncology focused on the assessment of the potential diagnostic possibilities of gene-expression profiling to serve as a more specific follow-up to the histopathological examination in controversial RCC cases. Different platforms have been used until now, including cDNA microarray (clones), Affymetrix technology (probe sets, genes), and nylon cDNA microarray (clones) (35–43). It has to be understood that although most of these studies differ much in terms of their methodological concept, their most significant findings coincide. For example, the gene most often up-regulated in hypoxia conditions and in the presence of VHL mutation is cyclin D1 (44).

Although the identification of genetic changes – as a result of advanced genomic studies – is undoubtedly a step forward toward personalized medicine in RCC, it should be noted that the exact and detailed role of most of these mutations still remain unclear. Subsequently, targeting these mutations could lead to many difficulties. However, such studies should be continued as there may still be other RCC subtypes waiting to be discovered.

## Potential Predictive and Prognostic RCC Histological Subtypes Biomarkers and Their Description

Molecular biomarkers are currently defined by the National Institute of Health Biomarkers Definitions Working Group (45). This organization was founded primarily to address the rapid expansion of gained (mainly genomic) data in the field of the diseases’ molecular basis (39). Ngo et al. have searched through PubMed for the phrase “renal cell carcinoma and biomarker” and have initially found over 3000 publications (46). Nonetheless, not all of those that came up in the search were really essential. It is known that, there are no standard approaches that can be used in biomarker sampling or in the analysis of kidney cancer. There are technical problems to be faced as well. For example, blood biomarkers are extremely vulnerable to many degradation processes (such as proteases, nucleases, and so forth).

Out of 48 studies analyzed by the Funakoshi scientific group, only three have satisfied Level 1 evidence for progression-free survival (PFS) in RCC. None of these is commercially produced in the market. These three are interleukin-8 (IL-8), hepatocyte growth factor (HGF), and osteopontin (47). This means that at present, no RCC biomarker is a fully credible candidate for use in clinical practice.

In the current literature, information on the definitions of the terms “prognostic” and “predictive” may sometimes be misleading. A prognostic biomarker is a biomarker that possesses a minimum of one clinical or biologic, objectively measurable characteristic. At the same time, a prognostic biomarker provides information on the probable outcome of a specific disease (in this case, renal cell carcinoma) in an untreated patient. Prognostic markers are widely used to identify individuals with cancer and who are at high risk of developing metastases. These markers also may serve as potential candidates for adjuvant therapy. On the other hand, there are predictive factors that show the patients the probable benefits they can obtain from the treatment. Significantly, prognostic factors pertain to the effects of the tumor characteristics on patients. Predictive factors, on the other hand, pertain to the effects of treatment on the tumor (48).

Contemporarily, there are several main methods used to perform mRNA expression profiling on the entire genome scale. These range from gene-expression microarrays or gene-expression serial analysis to differential display. With regard to array platforms, they may consist of purified cDNAs (spotted) or oligonucleotides (mostly photolithographic) (44). Some examples of genomic research in the field of renal cell cancer biomarkers will be described below. Gene-expression microarrays concerning RCC have also been successfully used to seek for immunomarkers as well (49).

Garraway et al. (50) has predicated a few key principles that may underlie translational cancer research and serve as a guide to the genomics-driven RCC research. One of them states that “molecular pathways involved in tumor survival and progression are often enacted by genetic alterations” (50). This is true in the case of the specific histological subtypes of renal cell carcinoma. It should be noted that there is no universal molecular marker for RCC. However, a few large genome-wide associated studies have shown recently that some nucleotide polymorphisms (SNPs) exist and that they *may* increase the patients’ risk of developing renal cell cancer. Some authors have meta-analyzed gene-expression arrays; others have conducted studies themselves. Each time these authors identify susceptibility loci (2p21, 11q13.3, 12p11.23, etc.) (51–54), which often show the high importance of such array studies, they provide molecular prognostic tests for future patients.

In 2007, Dalgin et al. performed a high-throughput microarray analysis on the RCC gene-expression profiles in order to specify the characteristics of the molecular markers of each histological subtype. Specific tumor markers were proposed by his scientific team. Microarray analysis was a key process in this field. Among 158 genes, ATPase, H^+^ transporting, lysosomal 56/58 kDa, V1 subunit B1 (*ATP6V1B1*); egl-9 family hypoxia-inducible factor-3 (*EGLN3*), Solute Carrier Family 25 [*SLC25A5* (adenine nucleotide translocator), Member 51], and *beta-tubulin* (*TUBB*), have been confirmed to be crucial (30). However, in similar studies, new genes have been also found to be important in RCC. These genes are all related to the critical processes underlying kidney cell transformation. Some of them are involved in excessive angiogenesis, escape from apoptosis, and cell adhesion or proteolysis (54).

Gene-expression profiling has unfortunately been somewhat limited to ccRCC, distinguishing its typical mutations, alterations, and biomarkers on the basis of genome-wide research. For instance, Dondeti et al. have performed an integrated analysis of such data for 54 sporadic ccRCCs and, as a result, they have identified the secreted glycoprotein stanniocalcin 2 (*STC2*) and the proteoglycan versican (*VCAN*) to serve as potential oncogenes in ccRCCs. Both are located on the long arm of the fifth chromosome. In their functional assays, *STC2* and *VCAN* were shown to promote cell survival (55). Older research was carried out in 2001 by Takahashi et al., who performed pioneering studies at that time to better understand the molecular mechanisms underlying ccRCC progression (56). This scientific group studied the gene-expression profiles of 29 ccRCC tumors by using 21,632 cDNA microarrays. As a result, alterations dedicated to most of the studied ccRCCs and which were unique to clinical subsets were also defined (ceruloplasmin, kininogen, lysyl oxidase, and the now well-known *VEGF*) (57). Later studies confirmed these results (58–60).

In another study on ccRCC biomarker candidates (61) with an integrated analysis of copy-number and expression profiles, the authors proposed 22 significantly overexpressed genes compared to those in healthy kidneys. Twelve of them were judged as proto-oncogene candidates in ccRCC with the use of high-throughput techniques. What is interesting is that they were also located in locus 5q35.3, apart from the constantly overexpressed gene on the long arm of the eighth chromosome, which is *MYC* (data further confirmed) (61). Many other mutations have been reported to be unique for ccRCC and such results have been obtained mostly via detailed genomic analyses or even large-scale sequencing. It is hard to mention them all in this article because of their large number (61–67), which only underlines the significance of high-throughput research in this field.

Another study performed by Schmidt et al. in 1997 comprising the genetic linkage analysis on hereditary papillary RCC. Mutations of *MET* proto-oncogene were shown to be present in its tyrosine kinase domain, which has classified HPRC for targeted therapy against *MET* and *VEGF* receptors (27). Two years later, the same scientific group reported that hereditary PRCC was characterized by multiple, bilateral papillary renal carcinomas. They also screened a large group of sporadic papillary renal carcinomas and many other solid tumors for various mutations in *MET* proto-oncogene. Summarizing these along with previous results, they presented such mutations in 17 out of 129 sporadic papillary renal carcinomas, but not in other solid tumors. What is striking is that even in 1999, molecular modeling studies suggested the importance of activating mutations and it was known that such phenomena tended to facilitate the transition to the active form kinases, which, in this case was *MET* kinase, leading to the formation of a different histological RCC subtype (27, 28). This was an example of early genomic approaches geared toward defining hereditary papilloma RCC biomarkers.

In 2013, a significant paper on DNA methylation profiling was published (28). DNA hypermethylation has been used to show distinctive profiles of chromophobe RCC and renal oncocytomas (5% cases). The authors utilized the Infinium Human Methylation 450 Beadchips technique. They identified 30 hypermethylated and 41 hypomethylated genes, differing its expression between chromophobe RCC and renal oncocytomas (*p* < 0.05), which could easily stand for a reliable histological biomarker after utilization in the future (34).

Currently, there is one more promising biomarker in RCC diagnosis – the interleukin 6 (IL-6) (48). IL-6 does not only play a significant role in systemic inflammation, but it also has a serious impact on the angiogenesis and signal transducer and activator of transcription 3 (*STAT3*). Renal cell carcinoma is known to produce excessive levels of IL-6 compared to other tumors (68). Unfortunately, IL-6 still has not fulfilled the minimum of Level I evidence for outcome prediction. There is a need to conduct further genomic research on this matter especially that up to now, IL-6 has only been revealed as a result of the ELISA tests (48).

The most important and promising among the up-to-date RCC biomarkers (both predictive and prognostic) are presented in Table 1 along with the genomic techniques used to find them. It has to be strongly noted that up to this day, none of the potential biomarkers described above or presented in Table 1 have been validated for clinical use (68). Unfortunately, not all research conducted in this matter are reproducible; therefore, as Sonpavde and Choueiri state in their recent publication, the utilization of the Clinical Laboratory Improvement Amendment (CLIA) seems to be inevitable to enhance reproducibility. Heterogeneity and toxicity, described in further chapters, seem to be the main challenge (69).

**Table 1:** **Chosen RCC prognostic and predictive biomarkers, which have been discovered as a result of high-throughput genomic research conducted up to date**.

Candidate RCC biomarkers – type	Candidate RCC biomarkers – name	High-throughput method used	Reference
Predictive	*VEGFR-3* polymorphism rs307826	SNPs (Single nucleotide polymorphisms), genotyping	Garcia-Donas et al. (70)
	*IL-8* phenotype 276TT	SNPs, genotyping	Xu et al. (71)
	*HIF-1*α^a^ phenotype 1790AG	RNA microarray	Choueiri et al. (72)
	*VHL* mutation/methylation^a^	DNA sequencing	Garcia-Donas et al. (70)
	*OPN*	Multiplex bead array	Zurita et al. (73)
	*VEGF*^a^	Multiplex bead array	Zurita et al. (73)
	*TRAIL*	Multiplex bead array	Zurita et al. (73)
	*VEGFR-2*	Multiplex bead array	Zurita et al. (73)
	*MET*	Comparative genomic microarray analysis (CGMA)	Albiges et al. (74)
	*CXCR7*	Gene-expression analysis of TECs	Maishi et al. (75)
	*PD-0332991*	Array comparative genomic hybridization (CGH) and gene expression	Logan et al. (76)
Prognostic	miR21/10b ratio	Deep sequencing data from TCGA datasets	Fritz et al. (77)
	(*HIC1*) CpG island methylation	Pyrosequencing	Eggers et al. (78)

**VEGFR-3*, vascular endothelial growth factor-3; *IL-8*, interleukin-8; *HIF-1*α, hypoxia-inducible factor 1α subunit; *VHL*, von Hippel-Lindau; *OPN* = *SPP1* secreted phosphoprotein 1; *TRAIL*, tumor necrosis factor-related apoptosis inducing ligand; *MET*, mammalian target of rapamycin; *CXCR7*, chemokine (C-X-C motif) receptor 7; *PD-0332991*, palbociclib, inhibitor of cyclin-dependent kinase 4/6; miR, micro RNA; *HIC1*, hypermethylated in cancer 1*.

*^a^Biomarkers are attributed either to the “predictive” or “prognostic” candidates on the basis of the information obtained from the current literature (if a biomarker is more often cited in PubMed as “predictive” – it is attributed to the “predictive” group)*.

## RCC Genomic Studies Revealed Impaired Signaling Pathways

Genomic studies tend to gather large amounts of data. Subsequently, pathway analysis has become a commonly used technique in cancer research. Recent publications on the Gene Set Enrichment Analysis (GSEA) technique have been published by Zeng et al. (79). This method was first introduced in 2003 for other tumor types; however, in this particular case, ccRCC was given focus. Such computational technique, the main aim of which was to define the specific features of signaling pathways rather than individual genes, combined with a functional enrichment set analysis tool, was shown to provide complete information on crucial altered processes in ccRCC. Published this year, the study comprises seven datasets with 110 ccRCC cases and 73 healthy kidney samples as a control. The study resulted in the revelation of 17 down-regulated and 12 overexpressed genes. Most of the pathways revealed by the GSEA were immune system diseases pathways (see: RCC genome and treatment), amino acids metabolic pathways, and carbohydrate metabolic pathways. The alterations of the Janus kinase/signal transducers and activators of transcription *(JAK/STAT)* signaling pathway (30 genes altered), as well as the cytokine-cytokine receptor interaction pathway (50 genes altered) (79, 80) were vital. The Database for Annotation, Visualization and Integrated Discovery (DAVID) enrichment analysis system was also used and it identified other important altered pathways in the ccRCC. These included the peroxisome proliferator-activated receptor (PPAR) signaling pathway (22 genes up- or down-regulated), ErbB signaling pathway (26 genes altered), and the insulin signaling pathway (42 genes altered). It has to be noted that due to the huge number of altered metabolic pathways, it was possible that ccRCC was a metabolic tumor (79, 80). It should be noted as well that this finding, if widely accepted by clinicians, would radically change the therapeutic approaches used in many of the ccRCC cases.

The *JAK/STAT* pathway is the main signaling mechanism for a wide variety of cytokines and growth factors in mammals. Its activation stimulates cell proliferation, differentiation, migration, and apoptosis; therefore, it is crucial in the process of cancerogenesis. The *JAK-STAT* pathway also transmits the information received from extracellular signals (through the receptors located in the membrane) straight to the target gene promoters in the nucleus. This way, it provides mechanisms for transcriptional regulation without second messengers. The *JAK-STAT* is a highly adapted, ligand-specific signaling pathway whose main function is to strictly control gene expression. If *JAK-STAT* is deregulated, gene over, or under-expression could take place (81, 82).

The PPAR signaling pathway is essential as well. *PPAR*-activated receptors are nuclear receptor proteins that act as transcription factors that regulate processes, such as the expression of genes, cellular differentiation, development metabolism, and tumorigenesis. It has recently been reported that *TGF-beta1* induces epithelial–mesenchymal transition (proven to exist in ccRCC) in NRK52E cells via *SMAD* and *PPAR*-γ pathway (83).

The ErbB protein family or epidermal growth factor receptor (*EGFR*) family is a family comprising four structurally related receptor tyrosine kinases. Pénzváltó et al. have performed a research focused on identifying resistance mechanisms against tyrosine kinase inhibitors (sunitinib, erlotinib, lapatinib, sorafenib, and gefitinib at the clinically administered doses), targeting the *ErbB/RAS* pathway in 45 cancer cell lines (also in renal cell carcinoma, as this pathway was often targeted in the latest anti-RCC therapy) (82).

Dalgin et al. (30) also revealed that the top-ranked pathways with significant influence on RCC development included the following: *MAPK* signaling pathway, G-protein coupled signaling pathway (and others connected with metabolism), and the immune response signaling pathway (30). Apart from *MAPK*, all results from 2014 confirmed the results obtained in 2007 (42). Mitogen-activated protein kinase is a crucial particle. It regulates the activities of several transcription factors and phosphorylates and activates the *c-Myc* or *MNK* (MAP kinase interacting serine/threonine kinase), which in turn phosphorylates the cyclic adenosine monophosphate (cAMP) response element binding protein (CREB). Mechanistically, by changing the activities of various transcription factors, the mutations of *MAPK* can lead to the altered transcription of genes that are significant to the cell cycle (82).

**Figure 1:**
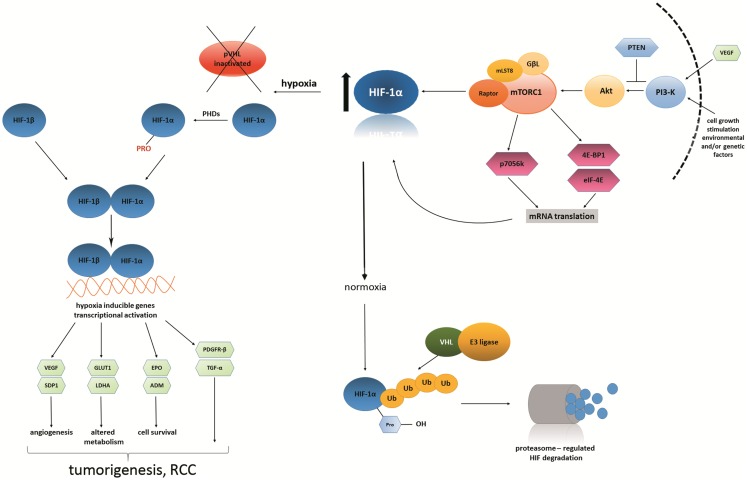
***Hypoxia inducible factor* and *VEGF* link the *HIF/VHL* and *PI3K/Akt/mTOR* signaling pathways**. Vascular endothelial growth factor, together with other external growth factors, activates *Akt*, which in turn activates mTORC1 complex The *PI3K* (phosphoinositide 3-kinase) pathway may be overactive because of faulty or deficient phosphatase and tensin homolog (*PTEN*). mTORC1 is a protein complex that functions as a specific controller of protein synthesis. It is composed of mTOR itself, a regulatory-associated mTOR protein named Raptor, mammalian lethal with SEC13 protein 8 (MLST8) and GβL, a positive regulator of the rapamycin-sensitive pathway required for the nutrient-sensitive interaction between Raptor and mTOR. What is striking is that it has been shown that mTORC1 complex, when up-regulated, subsequently up-regulates the expression of HIF-1α subunit (143–146). Under conditions of lower oxygen tension (hypoxia), VHL tumor suppressor protein becomes inactivated, which results in constitutive activation of the *HIF* pathway. HIF protein is heterodimeric; it consists of two constitutively expressed subunits: β-subunit and an oxygen-sensitive α-subunit. The latter is not degraded in such conditions; therefore, it translocates to the nucleus. Inside the nucleus, it undergoes dimerization with HIF-β subunit to form transcriptionally active *HIF*. In consequence, *HIF* as a transcription factor starts to regulate many biological processes via hypoxia-inducible genes, such as: *SDP1* (scan domain containing protein), *GLUT1* (glucose-transporter 1), *LDH* (lactate dehydrogenase), *EPO* (erythropoietin), *ADM* (adrenomedullin), *PDGFR-*β (platelet-derived growth factor-β), *TGF-*α (tumor growth factor-α). Other genes activated by mTORC1 complex: *p70S6 kinase, 4E-BP1* (4E-binding protein 1), *eiF-4E* (eukaryotic translation initiation factor 4E) (143–146). Under conditions of normal oxygen tension (normoxia), HIF-α subunit is hydroxylated by specific prolyl-hydroxylases and subsequently targeted for rapid proteasomal degradation. This is done by the VHL tumor suppressor protein, which is active at the time. In other words, HIF protein is degraded in proteasome when prolyl-hydroxylated α-subunits are targeted to the process of ubiquitination. It occurs by high-affinity binding to the VHL E3 ubiquitin ligase. Tumorigenic processes do not occur (147, 148).

With regard to the previously mentioned HIF subunits as well as *VHL* gene, it should be noted that for RCC, there is a *HIF/VHL* pathway that is mostly deregulated in one of its subtypes. Physiologically, the *VHL* gene is needed for HIF-1α degradation; therefore, in the case of *VHL* mutation (occurring in ccRCC), HIF-1α is not properly degraded and is subsequently overproduced. Overproduction of this subunit leads to the increase in the binding of HIF-1α to the so-called hypoxia responsive elements, which in turn causes the over-expression of the VEGF. VEGF is also significant in another crucial RCC pathway, the mammalian target of rapamycin (*mTOR*) pathway (84). The mechanistic target of rapamycin (serine/threonine kinase), mTOR is a protein encoded by the *mTOR* gene, a serine/threonine protein kinase that regulates cell growth, proliferation, motility, survival, and protein synthesis and transcription in humans. This pathway is named the *PI3K/Akt/mTOR* pathway and is essential in targeted RCC therapy concerning *mTOR* inhibitors (85). The detailed stages of these two combined pathways are shown in Figure 1.

To sum up, many crucial signaling pathways are impaired in renal cell carcinoma. Mostly, they are known in ccRCC. They are mainly connected with cellular differentiation (leading to tumorigenesis) or with altered metabolism. Research still needs to be done to explain the real meaning and consequences of the altered metabolism factor in the tumorigenic process of renal cell carcinoma.

## Is Personalized Therapy Really Possible? The Issue of Tumor Heterogeneity

In a recent systematic review of English-language literature with the use of the Preferred Reporting Items for Systematic Reviews and Meta-analyses (PRISMA) criteria, it has been clearly stated by Shuch et al. that contemporary understanding of renal cell carcinoma origin should switch from a uniform-malignant-phenotype model to a model in which heterogeneous group of cancers arise from kidney’s tubules (86).

It has recently been announced that the phenomenon of heterogeneity plays an important role in kidney cancer (87). In a recent study published this year, a new method called multi-region exome sequencing was used. The research that used this novel genomic tool revealed some substantial intratumoral heterogeneity in primary ccRCCs. This identification has significant potential for the future understanding of kidney cancer evolution and for developing effective therapies (87). In another review article published this year by Bex et al., the authors brought attention to the immunological heterogeneity of renal cell carcinoma microenvironment. Tumor heterogeneity is perceived nowadays as a key issue in molecular oncology (88).

There are four types of genetic heterogeneity in tumors according to Vogelstein et al. in their latest science publication (89). Each type is significant from the practical point of view when it comes to biomarker genomic research.

Intratumoral heterogeneity occurs among the various cells of one tumor. This phenomenon has been observed for years in science. Each time a cell divides, it acquires a specific amount of new mutations. Consequently, it is almost impossible to observe two genetically identical cells in one tumor (89). Lately, some studies have focused on the intratumoral heterogeneity evaluation of a genome (90–93). These studies mostly used the genome-wide sequencing technique. The common conclusion is that differences are meaningless in primary tumors as patients mostly undergo resection. Information obtained from such histopathological materials is crucial for understanding intermetastatic heterogeneity, especially among different metastatic lesions found in one specific patient (89). An interesting and highly probable hypothesis, which states that genetic mutations or other alterations that condition metastases formation occur earlier than the actual metastasis, has been proposed by Gerlinger et al. in 2012 (93).

There was also an intrametastatic heterogeneity among the cells of one metastasis of a patient, as well as an interpatient heterogeneity among the tumors of different patients. It was known that none of the oncologists had ever observed two genetically identical patients presented with the same specific cancer subtype) (94).

It has been brought to our attention by authors of several publications that most human cancers are caused by two to eight alterations occurring one after another and which tend to develop in the course of 20–30 years. During that time, formulating tumor tends to become genetically divergent as excessive cell division proceeds. Inevitably, genetic heterogeneity begins to form. It is always present and always influences the patient’s response to therapeutics; therefore, the most logical and most simple approach would be to perform genome-wide studies on the individual’s germline genome, along with the same research on the tumor. Such method of prevention would be a significant scientific achievement if successful (89, 91, 94).

Fisher et al. have already asked themselves a question, which is addressed in this chapter: “Is such heterogeneity a barrier to a personalized treatment in RCC?” Most probably, the concept that the sub-populations of different cancer cells (or of other cells) significantly affect the research and treatment of RCC is true. The most important problem concerns metastatic lesions. In reality, it is practically impossible to design a unique drug therapy for each individual patient despite ongoing research in the field of RCC biomarkers (95). There is no prognostic or predictive RCC biomarker for everyday use. It may be assumed that heterogeneity in RCC may significantly reduce the effectiveness of biomarker research. There is a need to improve and upgrade genomic studies to overcome difficulties or even find other possible ways to overcome the problem of heterogeneity using the knowledge of cancer genomics. The key step for researchers and clinicians and, most of all for patients, is the diagnosis. Early detection (in the first 10% of the cancer’s life span) almost guarantees 100% curability. Strong scientific, financial, and organizational efforts must be done to overcome the problem of heterogeneity to make RCC biomarkers enter the worldwide market (89). As microarray analyses have already resulted in the discovery of novel immunohistochemical markers, and several other markers have been validated to some extent (see Potential Predictive and Prognostic RCC Histological Subtypes Biomarkers Description), continuing this work is obligatory for combining such markers into specific diagnostic panels.

## Current State-of-the-Art and Recent Breakthroughs in RCC Genome and Treatment

Gene-expression profiles have been proven to be useful tools in identifying subtypes of cancer and in predicting outcomes. Prognostic and predictive biomarkers are currently under intense investigation. However, at this point in time, no validated gene-expression signatures for RCC are being used in everyday practice (96). Renal cell carcinoma, as a disease, lacks clinically applicative and validated predictive biomarkers for the most active therapeutic compounds, both targeting the mTOR (everolimus, temsirolimus) and the *VEGF* pathways (sunitinib, axitinib, pazopanib, sorafenib) (97). In some publications, the gene-expression profile has been judged as a poor predictor of disease free survival (DFS) or relapse/recurrence-free survival (RFS) (98).

A useful genomic tool in clinical RCC application, the 34-gene classifier (ClearCode34) has been developed and enabled lately to classify ccRCC tumors into two subtypes – referred to as ccA and ccB – with different RFS, cancer-specific survival (CSS), and OS (99). With the use of ClearCode34, the ccB subtype was characterized by the over-expression of various genes, including the Serpin Peptidase Inhibitor, Clade A Member 3 (*SERPINA3*), Solute Carrier Family 4 Member 3 (*SLC4A3*), Monooxygenase, DBH-Like 1 (*MOXD1*), Potassium Intermediate/Small Conductance Calcium-Activated Channel, Subfamily N, Member 4 (*KCNN4*), Receptor tyrosine kinase-like orphan receptor 2 (*ROR2*), Forkhead box M1 (*FOXM1*), and the UDP-*N*-acetyl-alpha-d-galactosamine:polypeptide N-acetylgalactosaminyltransferase 4 (*GALNT4*) gene. Hazard ratios (HR) for different ccRCC subtypes were similar to those in the clinical stage and grade in association analysis with recurrence risk. The HR for ClearCode34 remained significant in multivariate analysis as well. This gene-expression-based model was shown to be a better predictor for cancer-specific-survival compared to the widely used University of California, Los Angeles Integrated Staging System (UISS or UI), and Mayo Clinic Stage, Size, Grade, and Necrosis (SSIGN or SS) scores. ClearCode34 has demonstrated the superiority of the molecular model over the standard clinical predictive algorithm (99).

A novel gene-expression-based survival predictor was also published as a result of an even more advanced gene-expression analysis, which has covered nephrectomy cancer tissues obtained from 177 ccRCC patients. As a result, 259 genes were shown to accurately predict disease-specific survival (DSS) independently during the tumor stage, grade, and performance status. The authors of this research belonged to the Eastern Cooperative Oncology Group (ECOG). In the same analysis, the over-expression of 12 genes was associated with a shorter OS. These genes included the BCL2-Associated Athanogene 2 (*BAG2*), Guanine Nucleotide Binding Protein Alpha Stimulating Activity Polypeptide Secretogranin Complex Locus (*GNAS*), Immunoglobulin lambda constant 2 (*IGLC2*), and the Neutrophil cytosolic factor 1 (*NCF1*) (57).

Methodologically advanced genome-wide analyses were also performed in cases where tissues from primary RCC tumors and metastases were analyzed (94, 95). Pulmonary metastases that developed synchronously and metachronously were included in these analyses as well. Metastases had different expressions of 167 genes when compared to primary tumors. At the same time, 36 genes were shown to be differentially expressed when synchronous and metachronous metastases were compared (97, 98). In the study, gene-expression differences in the tumor and adjacent normal tissues from 93 patients have been detected using a genome-wide expression array. A panel of 661 inflammation-related genes was also analyzed. Consequently, various expression patterns between tumor and normal tissues were identified (97, 98).

This study was very important from the clinical point of view. The association of specific gene-expressions changed with the recurrence of the tumor and survival was evaluated on the mRNA and protein level. Prognostic significance was confirmed at the mRNA level for *CD31* (PECAM = Platelet/Endothelial Cell Adhesion Molecule 1), Endothelin receptor type B (*EDNRB*), and Tetraspanin 7 (*TSPAN7*). It was also confirmed at the protein level for *TSPAN7*. Patients with an over-expression of *EDNRB* and *TSPAN7* had significantly longer DFS and CSS. Patients with overexpressed CD31 had longer CSS only. In a multivariate analysis, only the *EDNRB* over-expression was confirmed as an independent prognostic biomarker of DFS. However, it is still not used in clinical practice, despite the fact that the over-expressions of *CD31, EDNRB*, and *TSPAN7* were defined as independent prognostic biomarkers at the CSS level (98). This analysis confirmed that the comparative analysis of tissues from the primary tumors and the metastatic samples may help to identify new prognostic biomarkers for RCC patients. The gene expression of the endothelial cells from the tumor samples should also be incorporated into future research so that there would be more possibilities of comparing the results with additional research control groups (98).

There are several other examples of genome-wide analysis concerning matters of RCC treatment. Recently, samples from 101 ccRCC patients have been used to identify genes that were differentially expressed in cancer tissues as opposed to paired adjacent normal tissues. Gene-expression data were used to identify biomarkers that were independently associated with OS. Statistical data covered the variables of age, sex, tumor grade, presence of synchronous or metachronous metastases, gene expression, and OS. This project identified eight genes, the over-expressions of which were associated with favorable ccRCC prognosis, one of which was epidermal growth factor receptor 5 (*EGFR5*) (100). In another trial, the over-expression of survivin (*BIRC5*) was shown to be associated with shorter CSS (101). A subsequent small study of 29 patients showed that the over-expression of 40 genes was a significant predictor of longer DFS and OS. The latter study differentiated between cohorts with 100% 5-year survival rate and patients with an average OS of 25.4 months and 0% 5-year survival rate (57). It was clearly visible that genomic research could produce valuable data in the clinical field concerning RCC as well.

Inflammation-related gene-expression profile was defined as a prognostic biomarker for the risk of ccRCC recurrence (for *GADD45G*: growth arrest and DNA-damage-inducible, gamma gene) and ccRCC death (for *CARD9*: caspase recruitment domain family, member 9, *CIITA*: class II, major histocompatibility complex, transactivator, and *NCF2*: neutrophil cytosolic factor 2 genes) (102). This was another example of the favorable use of genomic research as a method of choice in clinical applications. In this analysis, biological materials from 93 patients were analyzed using the genome-wide expression array, which included a large panel of 661 inflammation-related genes. Gene expression in the tumor was compared to the gene expression in the adjacent normal tissues. In the second step, the association of the gene expression with ccRCC recurrence (RR) and/or survival (OS) was analyzed. Among them, 151 genes that were differentially expressed with at least a twofold change were up-regulated in the tumor tissues. Of these genes, 20 were found to be significantly correlated with RR and/or OS. The over-expression of six genes, including the amyloid-β precursor, was associated with a prolonged period of recurrence (possible protective effect), referred to as a significant decrease in the recurrence risk (RR). At the same time, the over-expression of 12 other genes was associated with increased recurrence risk. These genes that were of high expression and that were associated with a higher risk of recurrence and shorter RFS included the B-cell linker, *CXCL1* [chemokine (C-X-C motif) ligand 1-melanoma growth stimulating activity, alpha], superoxide dismutase 2, cell surface associated mucin 1, and *CARD9*. In particular, the over-expression of *GADD45G* increased the RR by 2.09-fold while the risk of death was increased and OS was decreased alongside with the over-expression of *CARD9* (2.52-fold), *NCF2* (2.26-fold), and *CIITA* (2.11-fold). *CXCL1* over-expression was shown to result in the increased RR by 7.08-fold and in more than 30-month survival shortening. The most significant association for the increased risk of death was found in the adenosine A3 receptor (*ADORA3*) (HR 21.34) over-expression (102). On the other hand, for the interleukin 2 (IL-2) and IFN-α combination therapy, the over-expression of 14 genes was shown to predict a good response rate. Those genes included the *HLA* genes and the genes associated with immunological response, natural immunity, and cytokine expression (8, 103, 104).

Whole-genomic analysis was also used for treatment success. The analysis carefully covered the five selected patients with metastatic ccRCC and who were treated with mTOR inhibitor (everolimus or temsirolimus). This achieved a prolonged duration of stable disease (SD). This SD was greater on the rapalogs than on (first-line) the VEGF-targeted therapy (TKI). Patients with a median duration of 28 months were included in the study. Adjacent normal kidney tissue obtained at nephrectomy was used as control. DNA from tumors that matched normal tissues was analyzed using Illumina HiSeq 2000. In each individual patient, at least two regions from the same primary tumor were analyzed. If possible, a tumor tissue from a distant metastasis was also investigated. In this analysis, significant heterogeneity between primary and metastatic tumor and within a primary tumor was found. Benefit for the *mTOR* treatment was applied to functional alterations in two genes, namely, tuberous sclerosis 1 (*TSC1*) and mammalian target of rapamycin complex 1 (*mTOR1*) (96). Intratumoral heterogeneity was recently confirmed through gene-expression signatures. Good and poor prognosis were detected in different regions of the same tumor, but not in the same patient. This report also pointed to the activation of the *mTOR* kinase activity as significant for tumor growth and clonal evolution (93).

The European Union multi-disciplinary Personalized RNA interference to Enhance the Delivery of Individualized Cytotoxic and Targeted therapeutics (PREDICT) consortium started its activity in the ccRCC field over the last 5 years. This project was aimed at identifying the predictive biomarkers of sunitinib and everolimus treatment (96). To reach the objectives, two neo-adjuvant clinical trials were designed. These were the (1) S-PREDICT/PREINSUT for the sunitinib cohort and the (2) E-PREDICT for everolimus. The S-PREDICT/PREINSUT – biological, pathological, and imagery markers in the first-line treatment of metastatic clear cell renal cell carcinoma (NCT00930345) – was in phase four trial while E-PREDICT (SRCTN22979604) was in the phase one study of the everolimus treatment before nephrectomy in metastatic ccRCC. In those trials, fresh cancer tissues were collected before and after the 6-week-long treatment. Biopsies were taken before nephrectomy and were used as nephrectomy samples later on. Both trials were to recruit up to 60 patients from the discovery and validation cohorts. Treatment was stopped 2 weeks prior to nephrectomy. Responses were evaluated using the response evaluation criteria in solid tumors (RECIST) system. The authors claimed that the molecular genomic data may enable the individualization of ccRCC treatment and reduce ineffective therapy in drug-resistant individuals. A major focus in this project was to put on a small hairpin RNA (shRNA). Subsequent functional high-throughput small interfering RNA (siRNA) screens were used to identify DNA/mRNA drug response biomarkers and to point new drug targets for ccRCC. Another aim was to determine the sunitinib/everolimus acquired resistance mechanisms, as well as the response mechanisms of these drugs in susceptible subjects (96). MicroRNA expression was used as well in several other similar research studies (105, 106).

Emerging immunotherapies for renal cell carcinoma had been discussed by Escudier (107). Interleukin 2 (IL-2) and interferon alpha (IFN-α) were mentioned as the first immunotherapeutics. However, due to their high toxicity levels and the increasing evidence from the novel phase I/II clinical RCC trials, it was stated that new immunotherapies should be implemented into clinical use. They should be able to improve the patients’ outcome in a statistically significant manner. Currently, several vaccines are under intense investigation. These include (1) AGS-003, a dendritic cell-based vaccine, administered in combination with sunitinib in metastatic RCC; (2) IMA901, administered with cyclophosphamide and developed from HLA class I and II antigens; (3) MVA5T4, which stimulates the immune system to destroy 5T4^+^ cells, administered with sunitinib; and (4) autologous tumor cell lysate therapy. The modulation of the T-cells is possible and promising as well.

The role of immunotherapy for metastatic RCC should be considered again, particularly the possible role of genomics in novel immunotherapy solutions. Recent insights into the mechanisms of immune response in RCC patients, combined with high-throughput techniques, may allow a durable response to immunotherapy, perhaps even avoiding the toxicity that is usually associated with the previous types of immune treatment (107, 108).

At the end of this chapter, it is emphasized that no particular targeted drug designed specifically for the treatment of renal cell carcinoma as a result of genomic research has been accepted for clinical practice. With regard to the obvious potential of the genomic research, there is still more that needs to be done to implement the results mentioned above. Similarly, there is still more that can be produced via high-throughput genomic analyses.

## Conclusion

Knowledge on the molecular background of renal cell carcinoma constantly evolves due to many factors, such as the (1) advances in high-throughput technologies; (2) better understanding of the signaling and/or metabolic pathways involved in the crucial processes and affected by specific alterations; and (3) new targeted medical treatments (96). Genomic approaches have undoubtedly impacted the researchers’ and clinicians’ point of view in the field of renal cell carcinoma molecular characterization. Genome-wide studies have also provided enough data for the development of novel therapeutics for terminally ill patients according to their specific genotype. Still, there is more that needs to be done in this matter. More investigations are definitely necessary to determine which processes are critical for the induction and metastasis of kidney cancer-specific subtypes (109, 110).

It should be noted that the Food and Drug Administration of the US has not approved any microarrays for use in clinical practice even until now. This is due to several factors, including the following:

1. Quality control methods, as well as methods of data interpretation and analysis, have not yet been standardized. Until now, there are no specific and widely approved protocols for use in such studies (44).
2. There is a significant problem in the translation of genomic data into clinical practice (111).
3. Complex biomarkers that are currently under ongoing studies may bring both benefits and unknown risks (111).
4. Cancer heterogeneity and drug toxicity may be a specific barrier for the establishment of personalized therapy in renal cell carcinoma (95, 112).
5. Genomics-driven cancer medicine should always be rigorously evaluated, which is not possible in all cases (50).
6. Biological pathways are not known well enough to fully predict the relations between patient’s genotype and his or her outcome (109).

Why then is it necessary to conduct new genomic analyses and gather more data? Novel therapies for advanced renal cell carcinoma have substantially improved patient outcomes (113); however, specific patients’ genetic profiles linked with personalized treatment have not yet been discovered. Significant barriers to optimal care of renal cell carcinoma (RCC) definitely exist and those were mentioned by Rini et al. during the 2014 ASCO Genitourinary Cancers Symposium (113). During the examination and early treatment of RCC, it is basically not known whether the patient will respond to the specific targeted therapy. In the European scale and, for example, American scale, where complexity of targeted drugs implemented into practice is noticeable, there are many targeted drugs to choose from. The decision on which drug to choose is quite difficult as patients present different clinical benefit and have specific tolerance to, for example, targeted agents; up to date, the choice of therapy for an individual patient remains empiric (114). Moreover, targeted drugs have been found to be correlated with specific adverse effects. Some of them are known – for example in the case of sunitinib, which is a tyrosine kinase inhibitor (TKI) used for the treatment of RCC and gastrointestinal stromal tumor (GIST). It was reported that sunitinib is responsible for cutaneous adverse effects, such as hand-foot skin reaction (HFSR) in RCC and GIST patients (115). Still, many drug intake-adverse effect mechanisms remain elusive and genomic research may shed new light upon molecular biologists and oncologists knowledge in this field. Now, their main goal is to achieve a long-term phase of treatment benefit. To achieve prolonged survival, one has to keep in mind that again, despite obvious successes in RCC treatment, a number of patients still do not benefit from the therapy. Finding specific mutations which lead to adequate adverse effects in RCC patient is another obstacle and the reason for future research as well (87, 111).

There are improvements necessary to be made in other areas. For example, with regard to the patients’ survival, epidemiologic studies are also significant for genomic research acceleration (111). Here significant studies have been conducted – by Escudier et al. (116), Choueiri et al. (117), or Krabbe et al. (118).

Bioinformatic analyses need to be improved as well as current molecular biologists and biotechnologists are facing the problem on the management of individualized RCC genomic data. There are some useful databases available, such as My Cancer Genome; however, most of them are still under investigation (such as CollabrX or GeneInsight). With regard to hotspots – places with higher frequency of mutations – such databases are useful, but they still have to be upgraded with regard to the possibilities of combining data with treatment possibilities (119, 120).

Recent advances in the “-omics” era have shed new light for future clinical practice, prevention, detailed RCC classification, prognostic, and predictive markers development. New tools in genomics are slowly but constantly making it more possible to achieve. Large-scale genotyping, sequencing, and genome arrays also have a significant impact in studies concerning cancer tissues after nephrectomy or germ cells studies, not only on those conducted on stable cancerous cell lines. Some important successes in the field of genomic sciences in RCC genetic background have been reached (121, 122). For example, in the genome-wide association study (GWAS), Stadler et al. discovered RCC genetic variants in relation to its etiology (123). It is worth noticing that a few genome-based laboratory tests are already available (they mostly concern personal genome profiles) (110). More than 250 new tests were under investigation in 2010 (122).

It should be remembered that new technologies, such as next-generation sequencing (NGS), have enabled the systematic cataloging of RCC genomes, a crucial step needed to achieve the goal of overcoming the lack of clinical genomic applications. The first step is to relevantly assess clinical utility and validity. The second is to regard both not only in terms of hardware or software, but also in terms of the entire process of laboratory practice. The third is PFS (123). It is extremely crucial to educate healthcare professionals about genomics and its potential (124).

The challenge of discovering and subsequently implementing reliable RCC biomarkers are indisputable. Technical improvements in genomics have made the research easier despite the mentioned issues. For instance, a high-throughput sequencing technology is more cost-effective compared to other methods. On the other hand, sequencing the whole genome of germline DNA allows the identification of rare high-risk alleles for renal cell carcinoma (125). Studying such mutations in RCC may be possible in the future, especially if the multicenter networks of the patients’ molecular characterization are established. Such network has been created so far for lung cancer and is called the Lung Cancer Mutation Consortium (125, 126). Various methods of gathering new evidence in personalized medicine should lead to the building of a specific bridge between molecular laboratory and the clinic. This is possible to implement through the Rapid Learning Health Care (RLHC) model, which is very promising and is one of the key factors that can enable personalized genomic medical care (127, 128).

Each subtype of renal cell carcinoma, as well as its clear cell subtype, presents distinctive molecular and clinical features with unique tumor biology, patient prognosis, and patient’s response to treatment. With the new profiling techniques, we may now be able to analyze significant amounts of candidate genes not only to identify RCC subtypes, but also to improve prognostic and predictive information for patients and clinicians (94). Such research tools usage has been presented for example during the 2013 ASCO Genitourinary Cancers Symposium by Hakimi et al., who showed that microRNA may serve as novel blood-based biomarker in ccRCC diagnostic process (129). On the other hand, Luis M. Antón Aparicio et al. have presented their research project comprising molecular expression profiling and pathway analysis of formalin-fixed paraffin-embedded primary renal tumor specimens (130). Finally, also Hakimi’s research team has shown that the analysis of the Cancer Genome Atlas Project Association enables to gather a significant amount of information, in this case it were mutations in, previously mentioned in the article, chromatin modifiers, which positively correlated with poor survival in ccRCC (131). In fact, it has been suggested by Jones et al. that genomic research may provide information regarding the early stage changes in the gene expression in ccRCC, which may open the window for targeted therapy (111); especially that ccRCC is usually associated with the worst prognosis among other renal cancer subtypes. Unfortunately, none of the microarray data published clearly and fully identified the different panels of genes specific for each RCC stage. This may change in the future, though, if a team-based science, such as networks and consortia, would be applied. Combining knowledge obtained from different fields may lead to scientific improvements in personalized treatment in general (132).

In summary, it may be asked whether genomics could really serve as the first step toward personalized treatment in renal cell carcinoma. Fortunately, some studies conducted are repeatable (14, 101, 102) and have some considerable potential for the implementation of genomic research into clinical studies. There are no other techniques that can enable us to gather a huge amount of data than high-throughput analyses. The most important obstacle to overcome is adjusting to worldwide protocols, as a result of extended interdisciplinary teamwork, to transform those data into clinical practice (19). High-throughput research has one of the highest scientific potential that may be implemented into the research on renal cell carcinoma and this deduction is widely supported (19, 125–128).

Implementing personalized treatment is challenging not only in the field of renal cell carcinoma but also in the case of many other types of tumors; such approach would definitely require better understanding of cancer genomics and high-throughout methods at the same time. Understanding complex data sets obtained nowadays in huge amounts will be also necessary to achieve results in personalized care. From the molecular point of view, it is crucial that clinicians cooperate properly with molecular biologists or medical biotechnologists (or similar) in order to fully understand the issue of targeted drugs. The most appealing problem and therefore a challenge is obtaining such drugs and/or molecular markers based on the genomic profile of a patient’s tumor (133). It has to be clearly stated again that this issue concerns many cancer types; basically in most of them, genomic research have been conducted to seek for the opportunities of personalized care. Most up-to-date publications in PubMed in the field of cancer research with the use of genomic techniques include, i.e., RCC (134), lung cancer (135), hepatocellular carcinoma (136), breast cancer (137), colorectal cancer (138), leukemia (139), prostate cancer (140), etc. or complex studies (132, 141).

As it was stated by Renato Dulbecco, a co-recipient of the Nobel Prize in 1975, “the time has come to obtain a truly comprehensive catalog of genes involved in cancer, bringing to bear all the power of new tools of genomics and molecular biology to the problem” (142).

## Conflict of Interest Statement

Cezary Szczylik received consulting and lecture honoraria from Pfizer, Bayer HealthCare, Astellas, GlaxoSmithKline, and Novartis. Anna Małgorzata Czarnecka received lecture honoraria from Pfizer, GlaxoSmithKline, Novartis, Merck, Roche, Vipharm, and Hospira. All authors declare no other potential conflict of interests.
